# Hydrogen-rich water treatment targets RT1-Db1 and RT1-Bb to alleviate premature ovarian failure in rats

**DOI:** 10.7717/peerj.15564

**Published:** 2023-06-28

**Authors:** Xiaoyin Meng, Shuai Zhang, Lu Zhao, Yingmei Wang

**Affiliations:** 1 Department of Gynecology and Obstetrics, Tianjin Medical University General Hospital, Tianjin, China; 2 Tianjin Key Laboratory of Female Reproductive Health and Eugenics, Tianjin, China; 3 Tianjin Central Hospital of Gynecology and Obstetrics, Tianjin, China

**Keywords:** Hydrogen-rich water, RT1-Db1, RT1-Bb, Premature ovarian failure, Tandem mass tag (TMT)-based quantitative proteomic analysis

## Abstract

**Background:**

Premature ovarian failure (POF) is defined as the cessation of ovarian function before the age of 40 years, imposing a significant health burden on patients. However, effective etiological therapy for POF is scarce. Thus, we aimed to explore the protective role and targets of hydrogen-rich water (HRW) in POF.

**Methods:**

Based on cyclophosphamide (CTX)-induced POF rat models, the protective role of HRW treatment was mainly determined through serum 17-*β*-estradiol (E2), follicle-stimulating hormone (FSH), anti-mullerian hormone (AMH) levels, ovarian histomorphological analysis, and TUNEL assay. Tandem mass tag (TMT)-based quantitative proteomic analysis was then conducted on ovarian tissues, and the targets of HRW in POF were identified integrating differential expression analysis, functional enrichment analysis, and interaction analysis.

**Results:**

In HRW treatment of POF rats, the serum AMH and E2 levels significantly increased, and FSH level significantly reduced, indicating the protective role of HRW. After TMT quantitative proteomic analysis, a total of 16 candidate differentially expressed proteins (DEPs) were identified after the cross analysis of DEPs from POF vs. control and POF+HRW vs. POF groups, which were found to be significantly enriched in 296 GO terms and 36 KEGG pathways. The crucial targets, RT1-Db1 and RT1-Bb, were finally identified based on both protein-protein interaction network and GeneMANIA network.

**Conclusions:**

The HRW treatment could significantly alleviate the ovarian injury of POF rats; RT1-Db1 and RT1-Bb are identified as two crucial targets of HRW treatment in POF rats.

## Introduction

Various types of ovarian injuries have a significant adverse effect on patients (Chi et al., 2022; Cosgrove & Salani, 2019; Sims et al., 2022). Premature ovarian failure (POF), also known as premature ovarian insufficiency (POI), is defined as the cessation of ovarian function before the age of 40 (Tsiligiannis, Panay & Stevenson, 2019). It affects approximately 1% of women, with slightly varies among different ethnics (Szeliga et al., 2021). POF is characterized by menstrual disturbances (amenorrhea or sporadic menstruation), infertility, decreased estrogen and elevated gonadotropin (Qin et al., 2022). Unfortunately, the pathology of POF, except for the toxic impacts of galactose and its metabolites at ovarian level (Campbell et al., 2010), is still largely unclear, which leads to a lack of effective etiological therapies for POF women. Although clinical care, including hormone replacement therapy (HRT), follicle donation, and psychological support, is available for some POF patients, these patients are unable to have genetically-related children (Yan et al., 2018). Moreover, POF patients treated with HRT may suffer from a higher risk of coronary heart disease and breast cancer (Deady, 2004). Accordingly, it is important and imperative to explore novel protective and treatment means for POF patients, in order to improve their quality of life.

As the smallest molecule, hydrogen has been increasingly studied in many diseases serving as a promising therapeutic method, including cancers, metabolic diseases, and ischemia/reperfusion injury (Yang et al., 2020). Especially, since 2007, the protective role of hydrogen on multiple organs has been gaining accumulating attention (Ohsawa et al., 2007). The administration method of hydrogen is flexible, such as drinking hydrogen-rich water (HRW), inhaling hydrogen gas, etc (Liu & Qin, 2019). Hydrogen is able to penetrate biomembranes and diffuse into the cytosol, which thereby could be rapidly absorbed (Dixon, Tang & Zhang, 2013). Furthermore, high doses of hydrogen have not showed toxic effects as it can be exhaled *via* the lungs (Hong, Chen & Zhang, 2010), thus hydrogen displays valuable high bio-safety. In a cerebral ischemia-reperfusion rat model, HRW has been indicated to exhibit antioxidative, antiapoptotic, and anti-inflammatory effects (Lee & Choi, 2021). More recently, Ku et al. (2020) have evidenced that hydrogen-rich Korean Red Ginseng water is capable of increasing the production and motility of sperm through antioxidation and stimulating sex hormone. However, both the protective role of HRW on female infertility or POF and the potential targets of HRW have been rarely thoroughly investigated to our knowledge, seriously limiting its development in clinical applications.

Hence, the purpose of this study is to explore the potential effects and possible targets of HRW treatment on ovarian injury of POF rat models, in order to provide more reference information for the clinical application of HRW in the treatment of POF.

## Materials and methods

### Experimental Sprague Dawley (SD) rats

All adult, virgin, female Sprague Dawley (SD) rats (8–10 weeks old) were purchased from the Beijing HFK Bioscience Co., Ltd. (Beijing, China), and they were housed in the animal center of Tianjin Medical University (Tianjin, China). The animal experiments as well as humane care were approved by the Institutional Animal Care and Use Committee of Tianjin Medical University, in accordance with the National Institute of Health Guide for Care and Use of Laboratory Animals (protocol code: IRB2021-DW-16). All SD rats were housed under standard cycles (12 h light; 12 h dark) and specific pathogen-free conditions, with chow and water *ad libitum*.

### Preparation of hydrogen-rich water

The HRW was freshly prepared before the administration weekly, mainly according to the descriptions in previous report (Meng et al., 2015). Briefly, hydrogen was generated using a hydrogen generator (Beijing Zhongke Huiheng Technology, Beijing, China), and it was dissolved in physiological saline under 0.4 MPa pressure for 4 h. Then, the concentration of HRW was confirmed to be >0.6 mmol/L, employing a needle-type hydrogen sensor (Unisense A/S, Aarhus, Denmark). The HRW was stored in an aluminum bag with no dead volume at 4 °C, to ensure the eligible concentration of HRW.

### POF model construction and group settings

There were nine total SD rats in this work, and all rats were randomly divided into three groups, including the control group (con, *n* = 3), cyclophosphamide (CTX) induced POF group (POF, *n* = 3), and HRW treated POF groups (POF+HRW, *n* = 3). For the POF group and HRW treated POF group, all rats were given 50 mg/kg CTX (200–400 µL, NO.6055-19-2, Shanghai Yuanye Bio-Technology, Shanghai, China) by a single intraperitoneal injection to generate the POF rat model. Regarding control group, the rats received the equal volume of saline by intraperitoneal injection. In HRW treated POF group, the rats received intraperitoneal injection with HRW (10 ml/kg body weight) once a day, between the 1st and the 4th week. Meanwhile, the rats in control group and POF group were intraperitoneally injected with saline (10 ml/kg body weight).

Before sample collection, all rats were anesthetized by intraperitoneal injection of 3–4 µL/g 10% chloral hydrate. After sample collection, the rats were euthanized with carbon dioxide.

### Serum 17-*β*-estradiol (E2), follicle-stimulating hormone (FSH), and anti-mullerian hormone (AMH) levels determined by enzyme-linked immunosorbent assay (ELISA)

The blood samples in three groups were all withdrawn from the retro-orbital plexus of unconscious rats. The serum samples were obtained after centrifuging blood samples at 1800 r/min for 15 min. The E2 (NO. E-EL-0152c), FSH (NO. E-EL-R0391c), and AMH (NO. E-EL-R3022) serum levels were measured using corresponding ELISA Kits (Elabscience Biotechnology, Wuhan, China), and the protocols were strictly in accordance with manufacturer’s instructions.

### Ovarian histomorphological analysis

Before the paraffin embedding, three groups of ovarian tissues were firstly maintained in 10% buffered formalin for 48 h, which were then sliced (5 µm). Briefly, the tissue sections were then air-dried, dewaxed, then stained with haematoxylin and eosin (H&E). The slides were observed under Olympus BX40 light microscope (Olympus, Tokyo, Japan), and analyzed in ImageJ software. The numbers of primordial, primary, secondary, antral and atretic follicles were counted as previously described (Kamal et al., 2022).

### TUNEL assay

After the isolation of ovarian, the adipose tissues were eliminated from the ovarian. The ovarian tissues were then embedded in paraffin and sliced. The TUNEL assay was conducted using TUNEL Apoptosis Detection Kit (NO.11684817910; Roche, Basel, Switzerland), following the manufacturer’s instructions. Fluorescence microscope (Olympus, Tokyo, Japan) was used to observe the apoptotic cells. The proportions of apoptotic cells were calculated according to apoptotic cell counts/ total cell counts (five random fields per section and five sections per tissue from a rat).

### Ovarian tissue protein extraction and tandem mass tag (TMT)-based quantitative proteomic analysis

Briefly, the flowchart of the TMT-based quantitative proteomic analysis mainly involved protein extraction, trypsin digestion, TMT labeling, and liquid chromatography-tandem mass spectrometry (LC-MS/MS) data acquisition. Proteins of ovarian tissues were quantified using bicinchoninic acid (BCA) assay, and filter aided proteome preparation (FASP) was utilized for the trypsin digestion (Wisniewski et al., 2009). Subsequently, 100 µg peptides were taken from each sample (three biological repeats for each sample), which was subjected to the TMT labeling using TMT labeling kit as per the instructions of manufacture (Thermo Fisher Scientific, Waltham, MA, USA). Finally, the samples were processed using high pH reversed-phase high-performance LC (HPLC), and the raw data were assigned to the peptide sequences basing on the database comparison.

### Differentially expressed protein analysis

The differentially expressed proteins (DEPs) between various groups were analyzed using limma function of R (version 4.1.0, the same below). The DEPs with fold change <0.9 or >1.1, and *p* < 0.05 were considered statistically significant.

### Functional enrichment analysis

All significant candidate DEPs were then subjected to the Gene Ontology (GO) and Kyoto Encyclopedia of Genes and Genomes (KEGG) functional enrichment analysis, in “clusterProfiler” of R (Yu et al., 2012). Significant GO and KEGG terms were screened with *p* < 0.05.

### Subcellular localization prediction

The amino acid sequences can be converted into numerical localization features in WoLF PSORT database (https://wolfpsort.hgc.jp/) (Horton et al., 2007). The subcellular localization of candidate target proteins was predicted using a k-nearest neighbor classifier.

### Protein-protein-interaction (PPI) analysis

The STRING database (https://string-db.org/) integrated numerous known and predicted associations between proteins. Herein, we used STRING database (version 11.0) (Szklarczyk et al., 2019) to analyze functional associations and interactions between proteins.

Additionally, the candidate target proteins were analyzed using GeneMANIA database (http://genemania.org). GeneMANIA was a flexible database that predicted the function of genes/ proteins and prioritized genes for functional assays. For the given gene list, functionally similar genes would be identified *via* searching vast amounts of genomics and proteomics data. Then, GeneMANIA could weight each functional dataset according to the predicted value of the given gene list.

## Results

### Hydrogen-rich water alleviated ovarian injury of POF rat models

**Figure 1:**
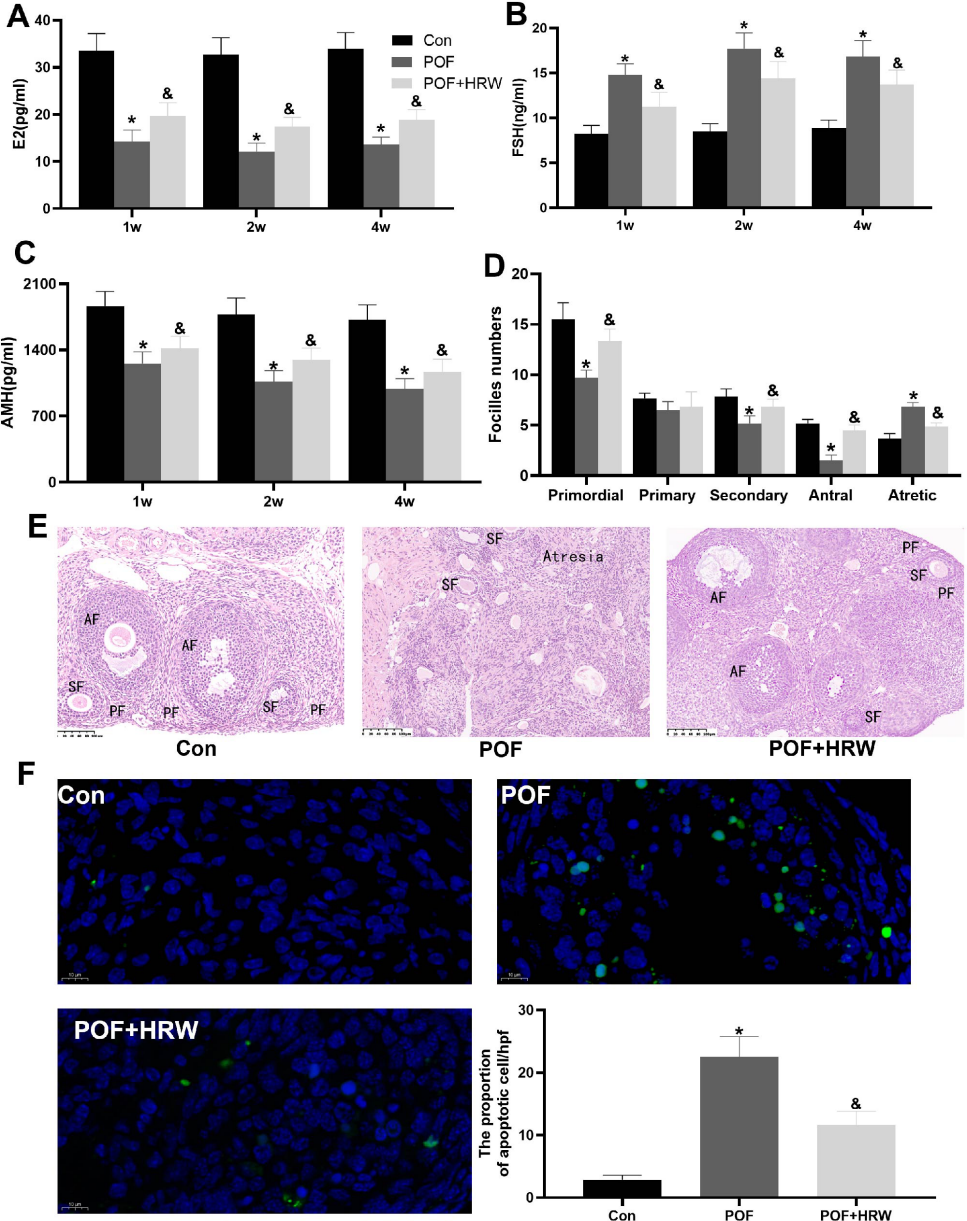
Protective effects of HRW treatment on the POF rats. (A–C) The serum E2 (A), FSH (B), AMH (C) levels were tested in three groups. (D) The numbers of follicles at different developmental maturation stages were counted in control group, POF group, and POF+HRW group. (E) Representative follicles in control group, POF group, and POF+HRW group were displayed after H&E staining. Scale bar: 40×. PF, primordial follicles; SF, secondary follicles; AF, antral follicles. (F) Apoptosis was analyzed using *in situ* TUNEL fluorescence staining and the number of apototic cells were counted and was compared among the three groups (*n* = 3). All data were shown as the mean ± s.d. Statistical significance: * *P* < 0.05, *vs* the control group, ^&^ *P* < 0.05, *vs* the POF group.

Firstly, we have constructed the POF rat models *via* CTX inducing. Compared to rats in control group, rats in POF group had markedly reduced serum E2 (Fig. 1A) and AMH (Fig. 1C) levels, while increased serum FSH (Fig. 1B) levels throughout the four weeks. After the determination of crucial hormone levels in serum, we confirmed that the POF rat models have been successfully built.

Next, we evaluated the effects of HRW treatment on POF rats. The rats in HRW treatment group had significantly increased serum E2 (Fig. 1A) and AMH (Fig. 1C) levels and significantly decreased serum FSH (Fig. 1B) level compared to POF group without HRW treatment. In addition, the primordial, primary, secondary, antral and atretic follicles in control group, POF group and POF+HRW group were counted after H&E staining, respectively (Fig. 1D). The representative HE images were displayed in Fig. 1E. Follicle counting results indicated that HRW treatment significantly affected the number of primordial, secondary, antral and atretic follicles in POF rats (Figs. 1D and 1E). Counting of primordial follicles showed that HRW treatment POF group significantly increased primordial follicles in POF+HRW group compared to POF group (Figs. 1D and 1E). Significantly more follicles were counted at different developmental maturation stages in POF+HRW group than in POF group (Figs. 1D and 1E). In the TUNEL assay, the numbers of apoptotic cells in ovarian tissues were assessed in all three groups, more TUNEL-positive cells were detected in POF group than in POF+HRW group and control group (Fig. 1F). Therefore, our results indicated that the HRW treatment alleviated ovarian injury of POF rat models.

### Candidate treatment targets of HRW in POF rat models

**Figure 2:**
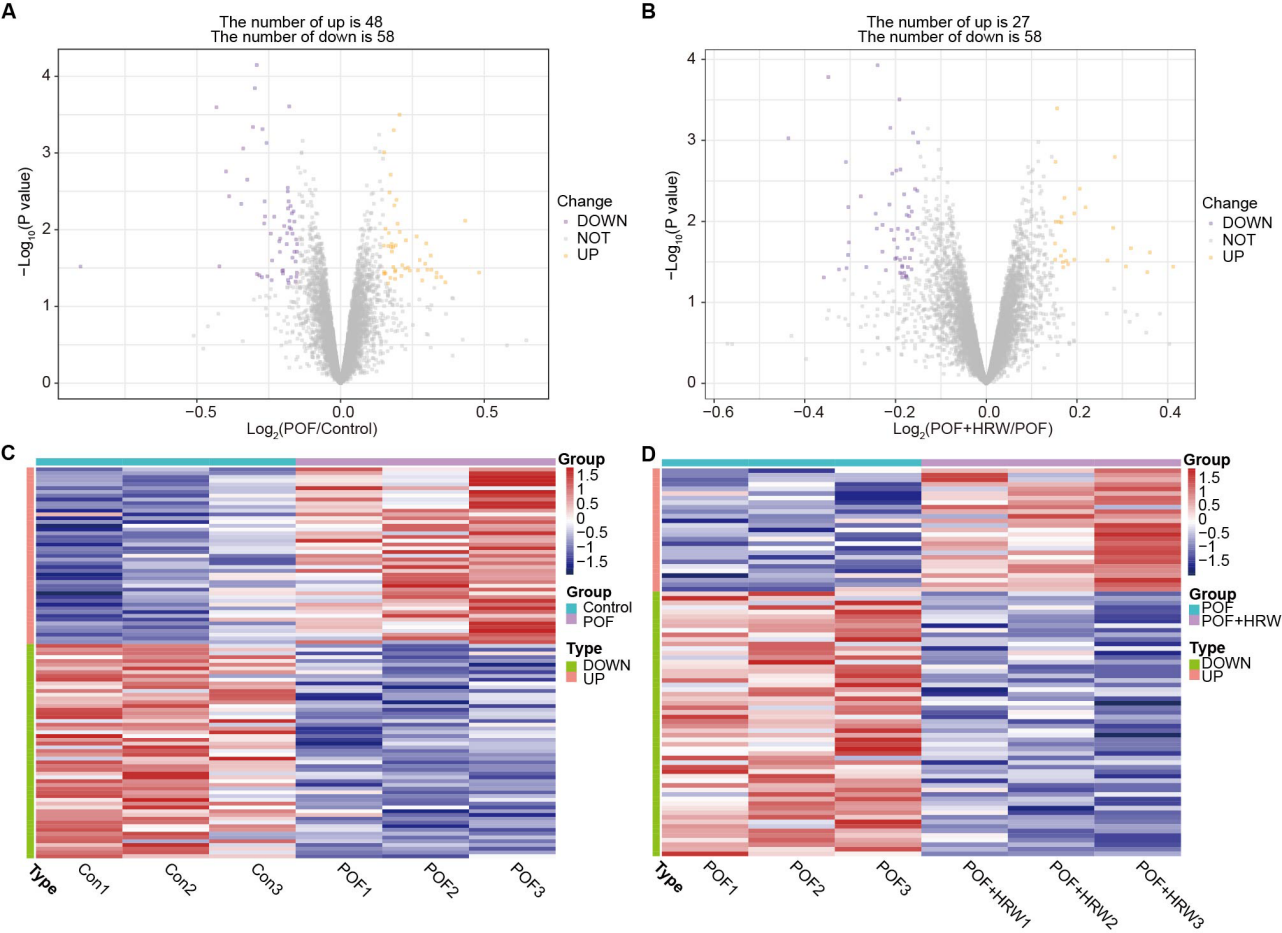
Candidate treatment targets of HRW in POF rat models. (A–B) The DEPs identified between POF *vs.* control and POF+HRW *vs.* POF, respectively. (C–D) The DEPs between POF *vs.* control, POF+HRW *vs.* POF exhibited significantly differential expression, separately.

Then, TMT-based quantitative proteomic analysis was performed on the ovarian tissue samples from three groups, in order to obtain the potential targets of HRW treatment in POF (raw data was provided in Table S1). Compared to control group, there were totally 106 DEPs in POF group, comprising 48 upregulated proteins and 58 downregulated proteins (Fig. 2A). In total, 27 proteins were upregulated and 58 were downregulated in POF+HRW group, compared with POF group (Fig. 2B). The DEPs between POF *vs.* control, POF+HRW *vs.* POF exhibited significantly differential expression, separately (Figs. 2C–2D). A total of 16 DEPs were then identified after the cross analysis of DEPs from POF *vs.* Control and POF+HRW *vs.* POF (Fig. S1, Table S1), which were important candidate targets of HRW treatment in POF.

### Functional enrichment results of candidate targets of HRW

**Figure 3:**
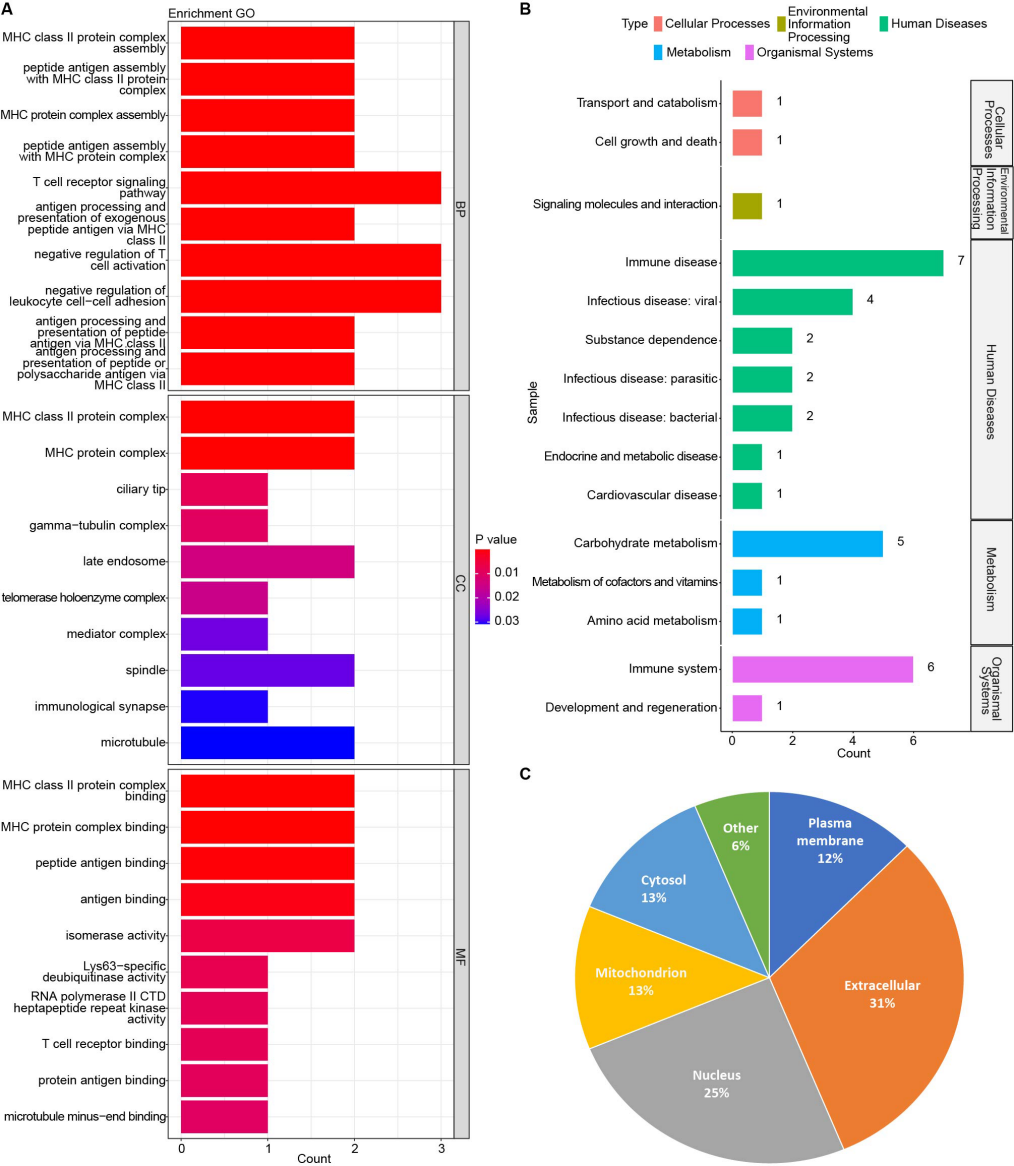
Results of GO and KEGG functional enrichment analysis. (A) Top 10 significant BP, CC, and MF terms. (B) The sub-type distribution of the 36 significant KEGG pathways. (C) The subcellular localization of the 16 candidate targets.

The GO and KEGG functional enrichment analysis was performed on the above 16 candidate targets to reveal the potential functions affected by HRW treatment. Our results suggested that these 16 proteins were significantly enriched in totally 296 GO terms (*p* < 0.05, Table S2), comprising 232 biological process (BP) terms, 17 cellular component(CC) terms, and 47 molecular function (MF) terms (top 10 sub terms were shown in Fig. 3A). The 36 significant KEGG pathways involved in immune disease (seven terms), immune system (six terms), carbohydrate metabolism (five terms), cell growth and death (one term), endocrine and metabolic disease (one term), and so on (Fig. 3B, Table S3).

Additionally, the subcellular localization of the 16 candidate targets was predicted using the WoLF PSORT database, indicating that these proteins were mainly localized to extracellular matrix (31%), nucleus(25%), cytosol (13%), mitochondrion (13%), and plasma membrane (12%) (Fig. 3C).

### RT1-Db1 and RT1-Bb were crucial targets of HRW treatment in POF rat models

**Figure 4:**
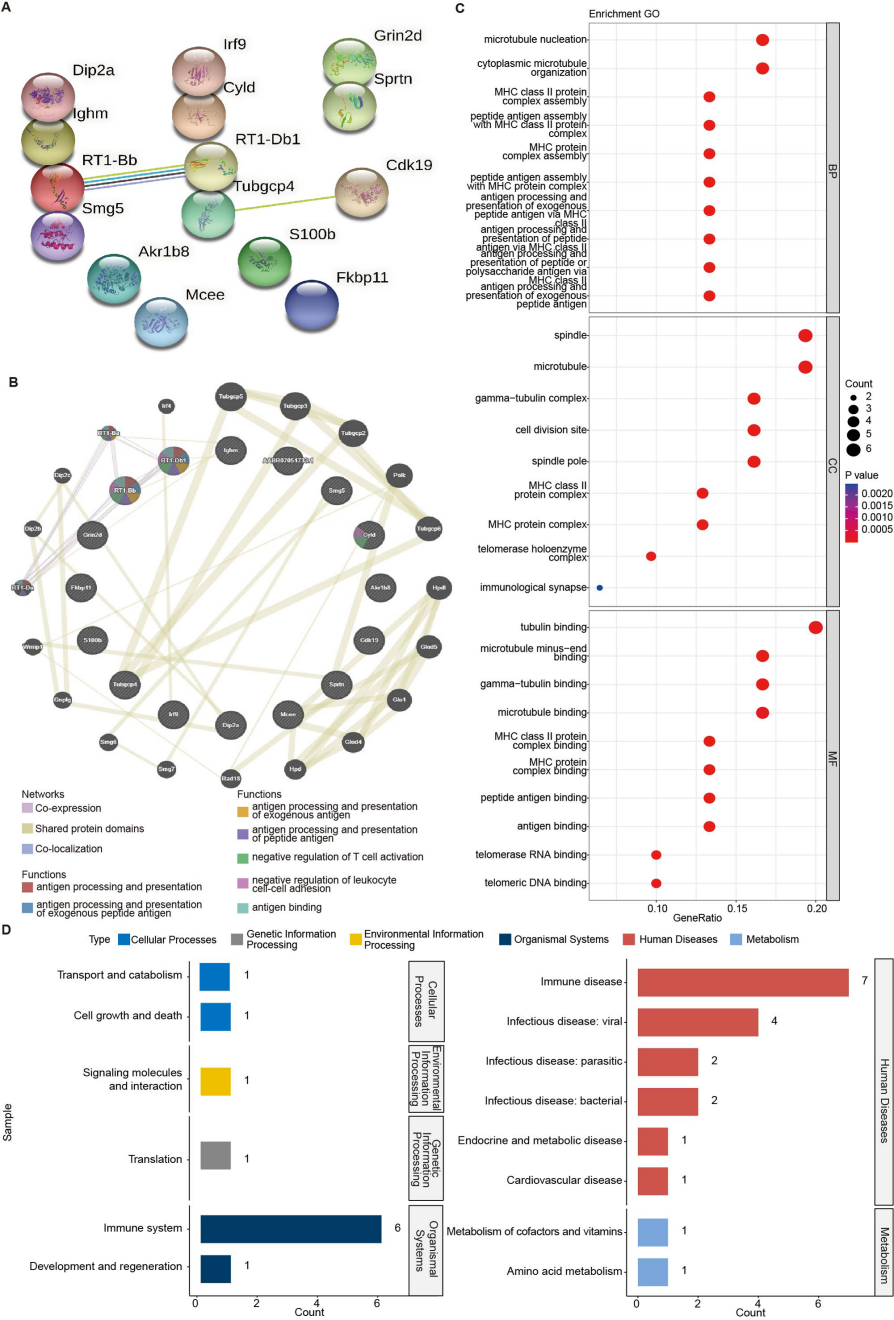
Interaction analysis of the candidate targets identified the crucial targets RT1-Db1 and RT1-Bb in HRW treatment PDF rats. (A) The PPI network based on 16 candidate targets. (B) The GeneMANIA interaction network based on 16 candidate targets. (C) Top 10 significant BP, CC, and MF terms. (D) The sub-type distribution of the 30 significant KEGG pathways.

We subsequently conducted the PPI analysis on these 16 candidate proteins, using STRING online tool. Among these proteins, there were 15 nodes and two edges (average degree = 0.267) (Fig. 4A), and the interactions between RT1-Db1 and RT1-Bb were notable. To further screen more reliable crucial targets of HRW treatment more reliably, the GeneMANIA network was also analyzed basing on the candidate targets. As shown in Fig. 4B, the GeneMANIA interaction network involved in co-expression, shared protein domains, co-localization, and the potential functions of the proteins. We found that significant co-expression was observed between RT1-Db1 and RT1-Bb, and they showed significant co-expression with RT-Ba and RT1-Da. The functions of RT1-Db1 and RT1-Bb were significantly correlated with the antigen processing. Based on all 16 candidate targets and their related interaction proteins in Fig. 4B, GO and KEGG enrichment results were in line with our former functional results (Figs. 4C–4D, Tables S4 and S5). The corresponding human orthologous genes of RT1-Db1 and RT1-Bb were HLA-DRB5 and HLA-DQB1, respectively.

## Discussion

Currently, heavy health burden and psychological stress make it imperative to explore effective protective treatment methods for POF patients (Yan et al., 2018). To our knowledge, we have firstly demonstrated the potential treatment targets, RT1-Db1 and RT1-Bb, of HRW in CTX-induced POF rat models. Our findings have evidenced not only the protective role of HRW against ovarian injury but also the crucial targets of HRW in POF, providing a promising treatment strategy for POF.

POF patients predominantly manifested with follicle dysfunction and ovarian atrophy (Melekoglu et al., 2018). CTX has been indicated to seriously damage to ovarian follicles, which is thereby usually used for the POF rat model induction (Lv et al., 2021). Herein, the POF rat models were built through CTX inducing. Significantly lower serum AMH and E2 levels, but higher FSH level have been observed in POF group in our present work. In POF patients, the serum FSH level has been usually elevated, while the levels of E2 and AMH are decreased (Rudnicka et al., 2018). Moreover, AMH has been widely known as an important indicator of ovarian reserve function, reflecting the ovarian follicle pool size (Yan et al., 2018). Lower AMH levels have been widely reported in the cases of normal menopause and ovarian aging (Seifer, Baker & Leader, 2011). All above evidences supported that CTX-induced POF rat model was successfully constructed. The subsequent HRW treatment suggested that the serum AMH and E2 levels were significantly picked up, FSH level was reduced, which implied the protective role of HRW in POF rats.

To explore the alleviating effects of HRW against POF in more detail, the TMT quantitative proteomic analysis was conducted on ovarian tissue samples from various groups. A total of 16 candidate DEPs were then identified after the cross analysis of DEPs from POF *vs.* Control and POF+HRW *vs.* POF, which were significantly enriched in 296 GO terms and 36 KEGG pathways. Notably, 11 major histocompatibility complex (MHC) class II protein complex related GO terms, such as MHC class II protein complex assembly, caught our attention. MHC class II protein expression deficiency would cause a compromise of CD4+ T cell development and increased autoimmunity, thereby leading to suboptimal T cell response and severe immunodeficiency (Chen et al., 2017; Santambrogio, 2022) These findings could be linked with the significantly enriched autoimmune thyroid disease pathway, as autoimmune and metabolic disorders are important non-genetic risk factors for POF patients (Chon, Umair & Yoon, 2021). It has been estimated that approximately 4–30% of POF cases resulted from the autoimmune-related diseases, among which, the most correlated group is thyroid-related disturbance (Kirshenbaum & Orvieto, 2019; Sharif et al., 2019). The 36 significant pathways we obtained mainly belonged to immune disease, immune system, and carbohydrate metabolism (pathway class II, Table S3). Accordingly, functional enrichment results uniformly indicated that the protective role of HRW against POF probably involved in immune system and metabolism dysfunction. However, specific functional details and the related molecular mechanisms needed to be further explored and confirmed.

Finally, among all candidate targets, two crucial targets of HRW treatment in POF rat models, RT1-Db1 and RT1-Bb, were identified based on both PPI network and GeneMANIA network. The RT1-Db1 and RT1-Bb were orthologous to human HLA-DRB5 and HLA-DQB1, respectively. HLA-DRB5 and HLA-DQB1 both belonged to MHC class II proteins. Meanwhile, the predominant functions of the targets were involved in multiple antigen processing and presentation processes, which was compatible with our enrichment results. Welsh et al. (2020) have recently reported that the lack of MHC class II accessory molecule HLA-DO was associated with susceptibility to autoimmune diseases. In our work, the RT1-Db1 and RT1-Bb expressions were significantly decreased in CTX induced POF rats, while picked up after HRW treatment of POF rats, which seemed that HRW was able to affect the expression of MHC class II proteins. Therefore, the HRW treatment probably attenuated the ovarian injury of POF rats *via* targeting MHC class II proteins, RT1-Db1 and RT1-Bb. Although rare recent reports have documented the role of MHC class II proteins in POF, in 1999, Arif et al. (1999) have demonstrated that autoantigenic presentation to T lymphocytes by HLA-DQ molecules with Asp57-beta-chains was a key pathogenesis of POF. Whereas, whether HRW exerted protective role on POF rats through regulating RT1-Db1 and RT1-Bb expression as well as affecting autoimmune was unable to be concluded. Underlying mechanisms behind the altered RT1-Db1 and RT1-Bb in POF rats received HRW treatment deserved deepening exploration.

Additionally, although we have revealed the crucial targets in the protective role of HRW treatment on POF rats, there were still several limitations in our present work. The crucial proteins, RT1-Db1 and RT1-Bb, were obtained employing TMT quantitative proteomic analysis. Whereas, there was a lack of *in vitro* validation of the two crucial proteins, which should be finished in our subsequent investigation in the near future.

## Conclusions

To summarize, we herein have shed light on the protective role of HRW against POF and revealed the crucial targets of HRW treatment, RT1-Db1 and RT1-Bb, for the first time. Moreover, HRW treatment probably attenuates the ovarian injury of POF rats through multiple MHC class II protein complex related pathways. Our findings are expected to provide alternative methods in making therapy strategies for POF patients.

##  Supplemental Information

10.7717/peerj.15564/supp-1 Supplemental Information 1 The Venn diagram between POF vs. Control and POF+HRW vs. POF

10.7717/peerj.15564/supp-2 Table S1 The 16 overlapped DEPs between POF vs. Control and POF+HRW vs. POF, and the raw data of TMT-based quantitative proteomic analysis

10.7717/peerj.15564/supp-3 Table S2 Detailed list of significantly enriched GO terms

10.7717/peerj.15564/supp-4 Table S3 Detailed list of significantly enriched KEGG pathways

10.7717/peerj.15564/supp-5 Table S4 Detailed list of significantly enriched GO terms basing on 16 candidate targets and their related interaction proteins

10.7717/peerj.15564/supp-6 Table S5 Detailed list of significantly enriched KEGG pathways basing on 16 candidate targets and their related interaction proteins

10.7717/peerj.15564/supp-7 Supplemental Information 7 The ARRIVE guidelines

## List of abbreviations

POF: Premature ovarian failure
HRT: hormone replacement therapy
HRW: hydrogen-rich water
SD: Sprague Dawley
CTX: cyclophosphamide
E2: Serum 17-*β*-estradiol
FSH: follicle-stimulating hormone
AMH: anti-mullerian hormone
H&E: haematoxylin and eosin
DEPs: differentially expressed proteins

## Additional Information and Declarations

### Data Availability

The following information was supplied regarding data availability: The raw measurements are available in the Supplemental Files.
