# Supplementary figures and images for: Hydrogen-rich water treatment targets RT1-Db1 and RT1-Bb to alleviate premature ovarian failure in rats
Source: PeerJ. 2023 Jun 28;11:e15564. doi: 10.7717/peerj.15564 (PMC10314742; doi:10.7717/peerj.15564)

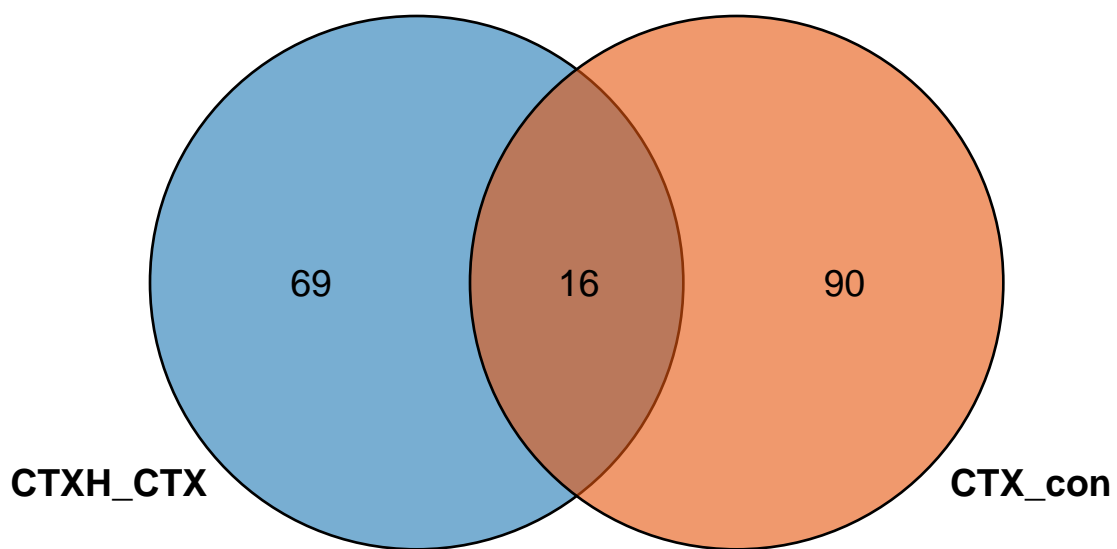

Supplement: Supplemental Information 1 [file peerj-11-15564-s001.pdf]
